# Potential determinant factors of under-five mortality in the Amhara region of Ethiopia

**DOI:** 10.1186/s12887-022-03253-x

**Published:** 2022-04-13

**Authors:** Nigusie Selomon Tibebu, Tigabu Desie Emiru, Chalie Marew Tiruneh, Adane Birhanu Nigat, Moges wubneh Abate, Bisrat Dessie Getu, Amsalu Belete Mekonnen

**Affiliations:** 1 Department of Pediatrics and Child Health Nursing, College of Health Sciences, Debre Tabor University, Debre Tabor, Ethiopia; 2 Department of Adult Health Nursing, College of Health Sciences, Debre Tabor University, Debre Tabor, Ethiopia; 3 Department of Nursing, Debre Tabor Health Sciences College, Debre Tabor, Ethiopia; 4 Department of Psychiatry, College of Health Sciences, Debre Tabor University, Debre Tabor, Ethiopia

**Keywords:** Under-five mortality, Amhara Region, Ethiopia, 2016 EDHS

## Abstract

**Background:**

Even though child mortality decreased greatly (44%, since 1990), children in developing countries are eight times more likely to die before they attain their five years birthday. When comparing under-five mortality around the world, the African including Ethiopia and Southeast Asian regions showed an uneven child death rate. Therefore, this study was aimed to identify the potential determinant factors of under-five mortality in the Amhara regional state of Ethiopia.

**Methods:**

Statistics from a national representative cross-sectional survey of the Ethiopian Demographic and Health Survey (EDHS) of the year 2016 were used. Data was collected from the population of all under-five children in randomly selected enumeration areas of the Amhara region of Ethiopia. To investigate the relationship between the dependent variable (under-five mortality) and various independent factors, inferential statistics such as binary logistic regression and multiple logistic regressions were used. In multivariable analysis, statistically significant variables in binary logistic regression analysis, i.e. (*p*-value 0.250), were entered, and *P*-value 0.050 was considered significant at 95% CI.

**Results:**

The survey was included 977 children under the age of five and more than half of children in the family (68%) were ≤ 4. The findings showed that children whose mothers had no formal education were 2.59 times more likely to die than children whose mothers had formal education [AOR: 2.59(1.12–5.99)]. Similarly, children who did not receive breastfeeding from their mothers were 3.61 times more likely to die than children who received breastfeeding from their mothers [AOR: 3.61(1.83–6.19)].

**Conclusion and Recommendation:**

The number of children in the family, as well as the mother’s educational status and current breastfeeding status, were all found to be important factors in under-five mortality in the study area. As a result, the potential determinants of under-five mortality should be addressed as part of a program targeted at lowering childhood mortality.

## Introduction

Despite falling short of the Millennium Development Goals (MDGs) targets, global child mortality has decreased by 44% since 1990. Children in impoverished countries, on the other hand, are eight times more likely to die before reaching the age of five owing to a variety of causes, including infectious diseases and poor nutrition [1]. Ethiopia is one of Sub-Saharan Africa’s countries with a high burden of child mortality [2]. In Ethiopia, the total mortality rate for children under the age of five was 67 deaths per 1,000 live births [3]. While, at the end of the Millennium Development Goals (MDGs), the under-five mortality rate was 59 deaths per 1000 live births [4, 5].

Under five Mortality (U5M) is higher in rural regions, and there is geographical diversity; the Afar region has the greatest death rate, with 125 deaths per 1000 live births, while Addis Ababa city administration has the lowest mortality rate, with 39 deaths per 1000 live births [3, 5].

The Sustainable Development Goals (SDGs) build on the successes of the Millennium Development Goals (MDGs) targets with the goal of emphasizing developmental science to other goals from birth to young adulthood in the sense of childhood health protection, which has had a significant impact on child mortality reduction [6, 7].

When comparing under-five mortality around the world, the African and Southeast Asian regions showed an uneven child death rate. Intending to end unnecessary deaths of children under the age of five, SDG 3.2 seeks to establish specific targets for child mortality [1].

A study on ‘breastfeeding and child survival’ was discovered in Bangladesh. Breastfeeding is an established predictor of mortality in children under the age of five [8]. Furthermore, the number of under-five children and breastfeeding were significant influencing variables of under-five mortality, according to Bangladesh DHS data from 2007 [9].

The under-five mortality rate varies widely across Africa’s sub-national levels. Additionally, research the essential tools for assessing sub-national progress toward the SDG child survival targets [10].

Maternal age, level of education, and religious background were all possible leading variables for U5M in Sub-Saharan Africa. Furthermore, maternal and child-related characteristics were discovered to be substantially correlated with U5M [11, 12].

According to a study conducted in Ghana using data from the 2014 DHS, the proportion of children under the age of five who die is 4.91%, while maternal education is identified as a key risk factor for the U5M rate [13, 14]. Breastfeeding status, mother education, and poverty were all key drivers of under-five mortality in a Nigerian study [15, 16]. Household poverty and breastfeeding were found to be predictors of under-five mortality in a study conducted in Kenya using data from the 2008–2009 Kenya Demographic and Health Survey [17].

According to the EDHS 2016 report, Ethiopia’s under-5 mortality rate was 67 deaths per 1,000 live births, but when compared to previous reports, all childhood mortality rates have decreased over time [3].

Despite the fact that a systematic study of childhood mortality in Ethiopia from 1990 to 2015 revealed a declining trend [4], socio-demographic [18], and socioeconomic factors [19] were identified as predictors of childhood mortality.

Between 2006 and 2011, there were 366 deaths before the age of five in Ethiopia’s high-mortality regions (specifically, Afar, Somali, Benishangul-Gumuz, and Gambela). Breastfeeding and the time between births were also likely determinants of childhood death [20]. The breastfeeding status of the infant and household members were determining variables of childhood mortality, according to a study conducted in Ethiopia’s Afar region on characteristics related to U5M [21]. Therefore, this study was aimed to identify determinant factors of under-five mortality in the Amhara Region of Ethiopia using the Ethiopia Demographic and Health Survey 2016 (EDHS) data.

## Methods

### Study area and setting

The Ethiopia Demographic and Health Survey (EDHS) was undertaken by the Ethiopian Central Statistical Agency (ECSA). The EDHS was funded by the Ethiopian government, the United States Agency for International Development (USAID), and other organizations. Aside from that, there’s a USAID-funded initiative that helps governments all around the world conduct demographic and health surveys by providing support and technical assistance. Ethiopia has a total of ten regional states. The study was conducted in Ethiopia’s Amhara regional state. The region’s enumeration zones were chosen at random, according to a report from the 2016 Ethiopian Demographic Health Survey (EDHS) [3].

### Study design and data source

The EDHS gives useful information on long-term trends in key demographic and health variables. The data gathered by the 2016 EDHS is designed to support policymakers and program managers in analyzing and designing programs and initiatives to improve the country’s population’s health. Therefore, the 2016 DHS dataset, which was collected country-wide from January 18, 2016, to June 27, 2016, was used to conduct a population-based cross-sectional analysis [3]. The information can also be found at https://dhsprogram.com/data/available-datasets.cfm. The data was analyzed using data from the 2016 EDHS, which included 977 samples.

### Inclusion and exclusion criteria

Parents and/or guardians of children under the age of five who were born within the five years prior to the poll were eligible to participate. Parents and/or guardians without U5 children, as well as those who were extremely unwell, were excluded from the study.

### Sample size determination

 For data analysis, 977 children with their parents and/or guardians were used. These figures are from secondary sources in the 2016 EDHS.

### Dependent variable

Under-five mortality is the study’s outcome variable.

### Independent variables

- Socio-Demographic Factors (education status of the mother, wealth status, sex of HH heads, residence, etc.)
- Maternal Characteristics (mothers age at first birth, birth type, place of delivery, ANC visit, etc.)
- Child’s Characteristics (sex of the child, size of the child at birth, numbers of children, breastfeeding, birth type, etc.)

### Statistical analysis

The data was presented and analyzed using descriptive and inferential statistics, respectively. To summarize the data, descriptive statistics such as frequency and percentage were used. Inferential techniques such as binary logistic regression and multiple logistic regressions were employed to investigate the relationship between the dependent variable (under-five mortality) and the various independent variables. In the binary logistic regression analysis, statistically significant variables (*p*-value < 0.25) were entered for multivariable analysis, with a *P*-value < 0.05 considered significant at the 95% confidence interval. The data were analyzed using the SPSS software suite.

## Results

### Socio-demographic factors of the participants

The analysis was included 977 children under the age of five. The majority of the participants (84.20%) were orthodox religious adherents. In terms of residence, the majority of the participants (90.90%) lived in a rural part of the region. Furthermore, when it came to the gender of the household heads, the majority of participants (91.00%) were male. The majority of the mothers were older than 16 at the time of their first birth (71.00%). When it came to the educational status of the children’s households, 732 (74.90%) of women had no formal education, while 719 (73.60%) had at least one ANC follow-up (Table 1).

**Table 1:** Characteristics of participants for under-five mortality in Amhara Region of Ethiopia (*N*=977)

Variables	Categories	Status of the child		Percentage (%)
		Dead (%)	Alive (%)	
**Socio demographic characteristics**				
Mothers’ education	No education	42 (4.29)	690 (70.63)	732 (74.92)
	Have education	7 (0.72)	238 (24.36)	245 (25.08)
Wealth status	Poor	24 (2.46)	427 (43.74)	451 (46.20)
	Medium	12 (1.22)	219 (22.38)	231 (23.60)
	Rich	13 (1.33)	282 (28.87)	295 (30.20)
Sex of the HH head	Male	42 (4.29)	846 (86.71)	888 (91.00)
	Female	7 (0.72)	82 (8.28)	89 (9.00)
Residence	Urban	4 (0.40)	85 (8.70)	89 (9.10)
	Rural	45 (4.60)	843 (83.30)	888 (90.90)
Religion	Orthodox	32 (3.28)	791 (80.92)	823 (84.20)
	Others^a^	17 (1.74)	137 (14.06)	154 (15.80)
**Maternal characteristics**				
Mothers age at first birth	< 16 years	16 (1.63)	267 (27.37)	283 (29.00)
	≥16 years	33 (3.38)	661 (67.62)	694 (71.00)
Place of delivery	Home	34 (3.48)	684 (70.02)	718 (73.50)
	Institutions	15 (1.53)	244 (24.97)	259 (26.50)
ANC	No visit	13 (1.33)	245 (25.07)	258 (26.40)
	Have visit	36 (3.68)	683 (69.92)	719 (73.60)
**Child characteristics**				
Sex of child	Male	33 (3.38)	468 (47.92)	501 (51.30)
	Female	16 (1.63)	460 (47.07)	476 (48.70)
Size of the child at birth	Larger	14 (1.43)	214 (21.87)	228 (23.30)
	Medium	15 (1.53)	405 (41.47)	420 (43.00)
	Smaller	20 (2.04)	309 (31.66)	329 (33.70)
Number of children in the family	≤ 4 children	40 (4.09)	629 (64.41)	669 (68.50)
	>4 children	9 (0.61)	299 (30.89)	308 (31.50)
Current Breastfeeding	Yes	20 (2.04)	688 (70.46)	708 (72.50)
	No	29 (2.97)	240 (24.53)	269 (27.50)
Birth type	Single	48 (4.91)	901 (92.19)	949 (97.10)
	Multiple	1 (0.10)	27 (2.80)	28 (2.90)

^a^Muslim, catholic

### Child’s characteristics of the study

Male children account for 51.3% of all children, which is nearly equal to female children (48.7%). Aside from that, the majority of children (72.5%) were breastfeeding (Table 1).

### Factors associated with under-five mortality in Amhara region of Ethiopia

To explore potential factors affecting under-five mortality, bivariable and multivariable logistic regression models were utilized. The sex of the household heads, the sex of the child, the mother’s education, current breastfeeding status, and the number of children in the family were all linked to U5M in a bivariable analysis with a *p*-value < 0.25.

The factors that were significant in the bivariable analysis were included in a multivariable logistic regression to adjust possible confounders. The number of children in the family, as well as the mothers’ education and current breastfeeding status, were all found to be strongly linked with U5M. However, with a *p*-value < 0.05, the remaining variables indicated above were not significant (Table 2).

**Table 2:** Bivariate and multivariable logistic regression analysis for Under-five Mortality in Amhara Region of Ethiopia (*N *= 977)

Variables	Categories	Status of the child					
		Dead (%)	Alive (%)				
				COR/95%/	*P*-value	AOR/95%/	*P*-value
**Socio demographic characteristics**							
Mothers education	No education	42 (4.29)	690 (70.63)	2.07 (0.92-4.67)	0.080	2.59 (1.12-5.99)*	0.036
	Have education	7 (0.72)	238 (24.36)	1		1	
Wealth status	Poor	24 (2.46)	427 (43.74)	1.22 (0.61-2.43)	0.576		
	Medium	12 (1.22)	219 (22.38)	1.19 (0.54-2.66)	0.678		
	Rich	13 (1.33)	282 (28.87)	1			
Sex of the HH head	Male	42 (4.29)	846 (86.71)	1	0.202	1	0.707
	Female	7 (0.72)	82 (8.28)	1.72 (0.75-3.95)		1.30 (0.55-3.08)	
Residence	Urban	4 (0.40)	85 (8.70)	1	0.812		
	Rural	45 (4.60)	843 (83.30)	0.88 (0.31-2.51)			
**Maternal characteristics**							
Mothers age at first birth	< 16 years	16 (1.63)	267 (27.37)	1.20 (0.65-2.22)	0.561		
	≥16 years	33 (3.38)	661(67.62)	1			
Place of delivery	Home	34 (3.48)	684(70.02)	0.81 (0.43-1.15)	0.514		
	Institutions	15 (1.53)	244(24.97)	1			
ANC	No antenatal visits	13 (1.33)	245(25.07)	1.01 (0.52-1.93)	0.987		
	Have antenatal visits	36 (3.68)	683 (69.92)	1			
**Child characteristics**							
Sex of child	Male	33 (3.38)	468 (47.92)	2.02 (1.10-3.74)	0.020	1.80 (0.94-3.26)	0.076
	Female	16 (1.63)	460 (47.07)	1		1	
Size of the child at birth	Larger	14 (1.43)	214 (21.87)	1			
	Medium	15 (1.53)	405 (41.47)	0.57 (0.27-1.20)	0.143		
	Smaller	20 (2.04)	309 (31.66)	0.99 (0.49-2.00)	0.981		
Number of children in the family	≤ 4 children	40 (4.09)	629 (64.41)	1	0.046	1	0.035
	>4 children	9 (0.61)	299 (30.89)	0.47 (0.23-0.99)		0.46 (0.22-0.99)*	
Current Breastfeeding	Yes	20 (2.04)	688 (70.46)	1	0.000	1	0.000
	No	29 (2.97)	240 (24.53)	4.01 (2.31-7.49)		3.61 (1.83-6.19)**	
Birth type	Single	48 (4.91)	901 (92.19)	1	0.724		
	Multiple	1 (0.10)	27 (2.80)	0.69 (0.09-5.23)			

1: Reference group, * *P*-value < 0.050(significant), ** *P *< -value 0.001(highly significant, *AOR* adjusted odds ratio, *COR* crude odds ratio, *CI* confidence interval

Children born to mothers with no formal education were 2.59 times more likely to die than children born to mothers with formal education [AOR: 2.59(1.12–5.99)]. Similarly, children whose mothers did not breastfeed had a 3.61 times higher chance of dying than children whose mothers did [AOR: 3.61 (1.83–6.19)]. However, children who lived in families with more than four children were 54% times less likely to die than children who lived in families with four or fewer children [AOR: 0.46(0.22–0.99)].

## Discussion

We looked at the factors that influence the mortality rate of children under the age of five, taking into report on variables from the literature as well as the dataset’s availability. The datasets from the 2016 EDHS were used to give in-depth information on the determinants of under-five mortality. According to reports, the country has seen a considerable decrease in under-five mortality in all regions. Nonetheless, the country has a higher risk of mortality among children under the age of five. According to the 2016 EDHS data, the Amhara region has the lowest under-five mortality rate, with 67 children dying per 1000 live births.

Even though different strategies and techniques have been used to reduce U5M, the current study found that mothers’ education, current breastfeeding status, and the number of children in the family were all found to be significantly associated with U5M in the Amhara region of Ethiopia.

Mothers’ education was one of the predictors of U5M in this study. Children whose mothers did not have a formal education level were 2.59 times more likely to die than children whose mothers had a high school diploma. The findings are backed up by a study conducted in Ethiopia DHS 2011 [18], Southern Ethiopia [22], Ghana [13, 14], Nigeria [16], Sub Saharan Africa [11, 23]. In truth, a mother’s education has been a positive effect on the living situations of her children. Furthermore, a well-educated woman increases the chances of her child’s survival by increasing the house’s and environment’s health through domestic sanitation and family planning.

The child’s breastfeeding status was also a factor in determining U5M. Children who did not receive breastfeeding from their mothers were 3.61 times more likely to die than children who received breastfeeding from their mothers. A study conducted in Ethiopia’s high U5M regions [20], Afar Ethiopia [21], Southern Ethiopia [24], Somalia Ethiopia [25], Kenya [17], Oyo State of Nigeria [15], Bangladesh [9], Matlab Bangladesh [26] backs up this conclusion. Breastfeeding has a significant impact on a child’s chance of survival since the mother’s milk guards them from infection [27]. As a result, children who have not been fed according to the recommended breastfeeding schedule are at risk, and their survival percentage will be reduced.

The number of children in the family was the last decisive factor of U5M in this region. Children who lived in families with more than four children were 54% times less likely to die than children who lived in families with four or less children. A study conducted in Bangladesh quote supports this conclusion [9]. The risk of death among children under the age of five is reduced when there are a large number of children (OR < 1). As far as we can determine, this conclusion is not supported by the evidence, so we’ve made a recommendation to researchers to investigate the depth of the relationship between number of children in the family and U5M using a high-level study design like a cohort. But, we suggest this might be because children are consuming things other than their mothers’ milk, and mothers will have more time to care for the remaining children.

### Limitations of the study

Even though the data was thought to be representative, the study has some flaws. It concentrated on children and maternal traits rather than environmental factors, and because it was a cross-sectional study, it did not prove a cause-and-effect relationship.

## Conclusions

The number of children in the family, as well as the mother’s educational status and current breastfeeding status, were all found to be important factors in under-five mortality in the study area. As a result, the potential determinants of U5M (breastfeeding campaign, and health education for mothers/parents) should be addressed as part of a program targeted at lowering childhood mortality. Additionally, we recommended researchers study environmental factors and child death in the Amhara region.

## Data Availability

The datasets used and/or analyzed during the current study are available in the Demographic and Health Surveys (DHS) [http://www.dhsprogram.com/data/available-datasets.cfm.]

## Abbreviations

ANC: Antenatal care
AOR: Adjusted odds ratio
CI: Confidence interval
COR: Crude odds ratio
CSA: Central Statistics Agency
EDHS: Ethiopian Demographic and Health Survey
MDGs: Millennium Development Goals
SDGs: Sustainable Development Goals
SSA: Sub-Saharan Africa
UNDP: United Nations Development Program
UNICEF: United Nations International Culture and Education Fund
U5M: Under-five mortality
WHO: World Health Organization
